# Status Quo and Research Trends of Neurosurgical Departments in China: Bibliometric and Scientometric Analyses

**DOI:** 10.2196/25700

**Published:** 2021-07-05

**Authors:** Bowen Ni, Minyi He, Bei Cao, Jianmin He, Yawei Liu, Zhen Zhao

**Affiliations:** 1 Department of Neurosurgery Nanfang Hospital Southern Medical University Guangzhou China; 2 Guangdong-Hong Kong-Macao Greater Bay Area Center for Brain Science and Brain-Inspired Intelligence Southern Medical University Guangzhou China; 3 Clinical Medicine Education Center Nanfang Hospital Southern Medical University Guangzhou China; 4 Laboratories & Facilities Management Office Southern Medical University Guangzhou China; 5 Institute of Scientific Research Southern Medical University Guangzhou China

**Keywords:** neurosurgery, bibliometric analysis, co-word biclustering analysis, visualized analysis

## Abstract

**Background:**

Modern neurosurgery is a relatively young discipline characterized by finesse and complexity. In recent years, neurosurgery in China has made continuous developments, with long-term progress and outstanding discoveries in many aspects of the field.

**Objective:**

This scientometric investigation aimed to comprehensively provide insight into the development trends of neurosurgery in China, to demonstrate how the field has evolved.

**Methods:**

PubMed database was searched to retrieve relevant papers published between 1988 and 2018 from neurosurgery institutions in China. The database of the National Natural Science Foundation of China was also retrieved for funding information. Information (eg, year of publication, journal, institute of origin) and keywords were collected from each paper after removing duplicates and filtering unintentional words. Co-word analysis was performed on the papers’ keywords, and a time distribution matrix of coexisting keywords in a given paper (ie, termed co-words) was established. Co-words were clustered according to their growth rate within years and visually presented with a mountain plot and a heatmap. Trends and potential subspecialties were identified, and each topic, represented either by a co-word from publications or funding from the National Natural Science Foundation of China during the period from 2011 to 2018, was collected and analyzed.

**Results:**

Within 15,972 publications on neurosurgery from institutions in China, diagnostic image was found to coexist the most with other keywords. Cluster 0, represented by diagnostic image with retrospective study, contained emerging topics with great developmental potential and demonstrated high growth rates in recent years. This finding suggests that the topics represented in Cluster 0 may represent future areas of important neurosurgical research. We also found that the developmental trend of China’s neurosurgical research is highly correlated with National Natural Science Foundation of China funding acquisition.

**Conclusions:**

Co-word analysis and visualization results provided insight into the emerging research topics that are of vital importance, which can be used as a reference by neurosurgeons and researchers for future investigations. In this study, our analysis strategy based on co-word biclustering was able to clearly demonstrate current academic subject development; therefore, co-word biclustering is a reliable bibliometric analysis strategy.

## Introduction

Modern neurosurgery, with a history of only 100 years, has been developing rapidly since its birth in the late 19th century. In past decades, modern neurosurgery has experienced three stages of development: classical neurosurgery, microneurosurgery, and minimally invasive neurosurgery [1]. Modern neurosurgery can be divided into neurotrauma, pediatric neurosurgery, functional neurosurgery, cerebrovascular disease, skull base lesions, brain tumors, spinal neurosurgery, and other subdisciplines [2]. After 1949, modern neurosurgery in China sprouted, and though it started later than it did in other countries, it has shown amazing growth, with an abundance of published papers [3].

For decades, the most common way for researchers to become familiar with the state of the art and development trends in research has been to read published reviews. However, the large amount of literature that can be found for a single topic makes it increasingly difficult for researchers to quickly find the areas of research they are interested in. In recent years, bibliometric analysis has become increasingly popular. It uses bibliometric characteristics, such as country, institution, journal, or author, to measure the contribution of a research field and to predict, in detail, the research trends or hotspots in a certain field [4,5].

Co-word analysis of keywords in a certain field can be performed, not only to gather detailed information about subdisciplines, but also, to detect intrinsic associations between them [6]. If keywords coexist in a paper, it is likely that the topics that they represent are related, to some extent [7]. Moreover, if a large number of papers contain coexisting keywords (ie, termed *co-words*) during a period of time or if the number of published papers has increased over time, it can represent an emerging area of interest that is currently, or will be in the future, highly important [8].

Cluster analysis, in particular, can help identify relationships between research topics. Bibliometric analysis has been used to identify the most influential papers in medical disciplines and specialties, such as gastric diseases [9], infectious diseases [10], and urinary diseases [11]; however, to date, literature trends from neurosurgery institutions in China have not been evaluated. We aimed to apply bibliometric and scientometric analysis techniques, such co-word matrixes and biclustering, to gain insight into domestic research on neurosurgery and subdisciplines, their evolution in recent years, and future development trends in China.

## Methods

### Search Methodology and Strategies

**Figure 1:**
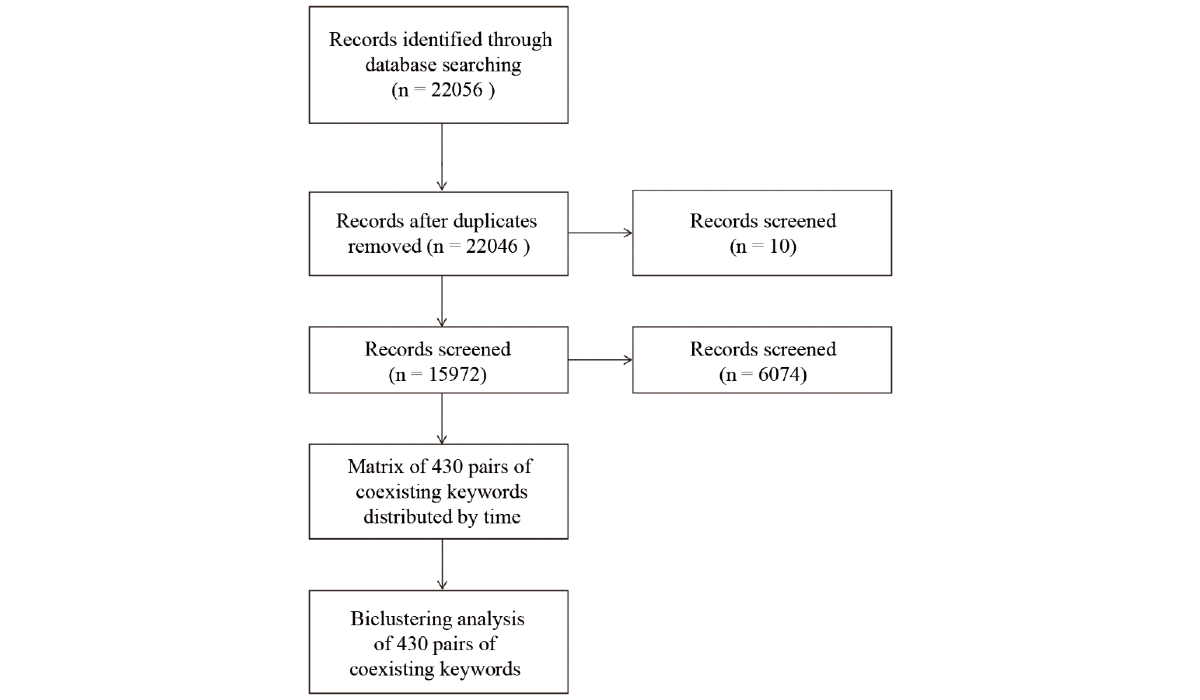
Data collection flowchart.

Published literature in the PubMed database was searched using the following search string: ((China [Affiliation]) AND neurosurgery [Affiliation]) AND (“1988” [Date - Publication]: “2018” [Date - Publication]). We performed the search on September 8, 2019, which yielded a total of 22,056 papers. Search results were screened to removed duplicates. We excluded articles about other subjects of study by title, abstract, and keywords (Figure 1). Article information, including the title, name of the journal, institution, year of publication, and keywords, was exported using the PubMed tool and saved as a document in .nbib format for subsequent analyses.

### Data Processing

We imported bibliography data and keywords from the papers using NoteExpress (version 3.3; Beijing Aegean Software Company), and Excel (version 2019; Microsoft Inc) was used to establish a database containing bibliography information, such as the year of publication, influencing factors, and journals, and perform descriptive statistical analysis. The main premise of bibliometric analysis based on keywords is that keywords selected by paper authors fully and accurately describe the main content of the paper [8]. If a paper’s keywords could not be extracted automatically by the software, we manually obtained the keywords from the paper in the PubMed database. If keywords were not available from the original source, we extracted subject headings from the title of the paper instead. The quality of keywords is of vital importance to the results of the subsequent analysis [12]. We standardized extracted keywords by (1) merging words with the same meaning, (2) removing duplicates, (3) changing plural to singular form, and (4) deleting unintentional words (such as numbers).

### Co-Word Analysis

After processing, there was a total of 15,972 keywords. Because there were relatively few papers published before 2010, the period from 1988 to 2018 was divided into 11 stages (1988-2000, 2001-2005, 2006-2010, 2011, 2012, 2013, 2014, 2015, 2016, 2017, and 2018). Distribution features of all keywords for every stage were quantitatively assessed for co-word analysis. We used data-mining software (BibExcel [13]) to calculate the frequency of every co-word (pair of coexisting keywords appearing in each paper) to form a matrix [14]. To understand the status quo and developing trends of co-words over time, co-word matrixes for every period were imported into R software (version 3.6.1; R Foundation for Statistical Computing), and a binary temporal distribution matrix, with co-words as rows and stages of time as columns, was constructed and exported with a merging algorithm—the number in each cell represented the cumulative frequency of each pair in a given time stage. The growth rate of each co-word pair (change in the number between successive stages) was calculated to analyze development trends of every research topic represented by each pair, and 430 co-words with a sustained growth trend (growth rate over 0) in 2015 to 2016, 2016 to 2017, and 2017 to 2018 were identified.

### Biclustering Analysis of Coexisting Words

gCLUTO software (version 1.0; Kerapis Lab) and OpenGL-based mountain visualization were used for biclustering analysis of co-words. We imported the binary matrix data of the 430 co-words into gCLUTO, which uses a clustering algorithm based on partitional clustering, to determine the best number of subgroups *k* based on height, color, and independence of each peak in the mountain plot.

The co-words were divided into 3 clusters, and co-word matrix data were shown by a peak plot and a heat map, which visually display distribution characteristics. Word cloud plots for the 3 clusters co-words were created, in which the size of each co-word was determined by its cumulative frequency. We also imported the co-word matrix into Cytoscape (version 3.8.2; National Institute of General Medical Sciences of the National Institutes of Health) to more intuitively reflect the relationships among 430 co-words with a circular layout.

### Topic Trends Between Publications and Funding

The National Natural Science Foundation of China is the most important funding source in China; therefore, to provide insight into the role played by funding in development trends, funding amounts from 2011 to 2018 for each subdiscipline represented by co-words were collected, and Pearson correlations were calculated.

## Results

### Basic Characteristics of the Papers

**Figure 2:**
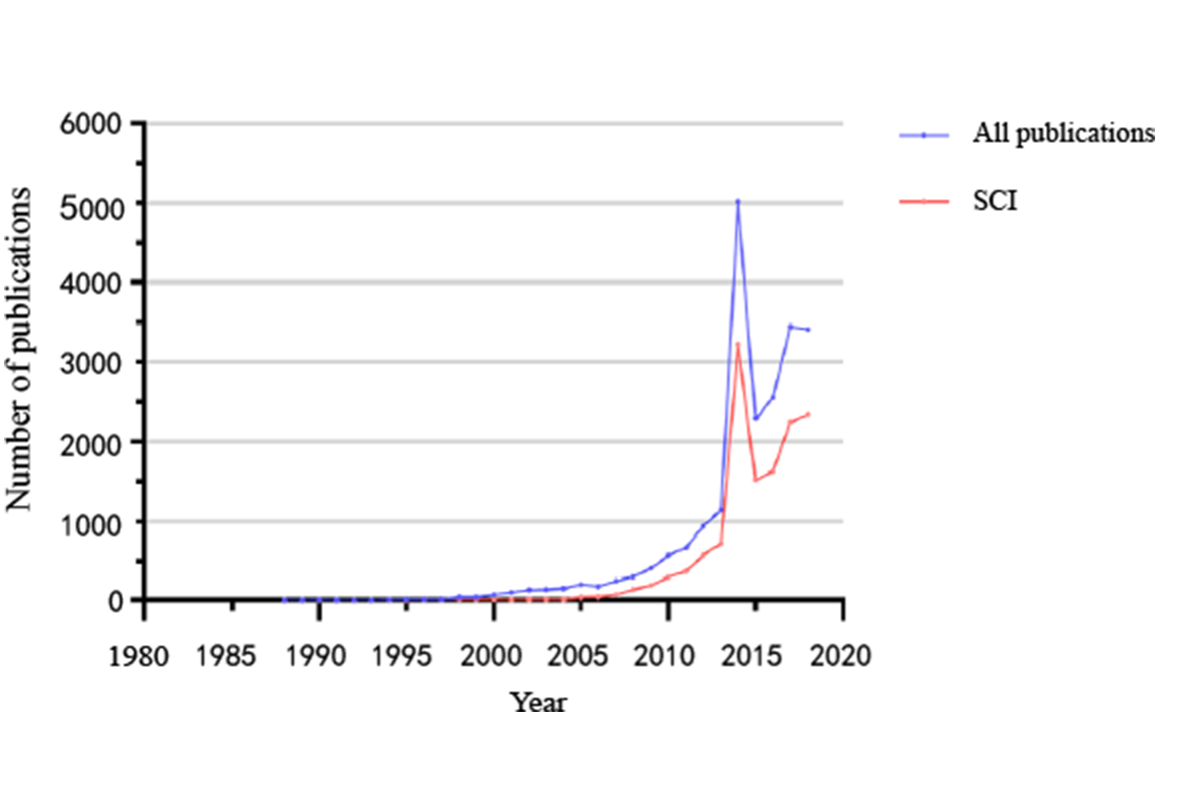
The temporal distribution of articles. SCI: Science Citation Index.

Changes in the number of publications in a certain discipline can be examined, not only to reflect development, but also, to predict future developmental trends. To understand the development of neurosurgery in China in recent years, 15,972 papers from 1988 to 2018 were statistically analyzed year by year (Figure 2). The results showed that the amount of papers have increased year by year and reached a peak in 2014 (5021/15,972, 31.44%). The amount of papers included in the Science Citation Index (SCI), accounting for 61.00% (9743/15,972), has also been increasing rapidly, which is demonstrated by an upward trend since 2007.

### Journal Distribution

Papers published by neurosurgical researchers in China appeared in 1420 journals, of which 802 are included in the SCI. The average number of papers published in the top 10 journals (Table 1) was 498, which was 32 times the number published in all other journals. *World Neurosurgery* featured the most papers (1096/15,972, 6.86%), followed by the *Chinese Medical Journal* (561/15,972, 3.51%) and *PLOS One* (481/15,972, 3.01%). Of the top 10 journals, 5 were included in the SCI, among which *PLOS One* had the highest impact factor (2.776). In total, 2906 papers were published in journals in the SCI (2906/4981, 58.34%), including those in the top 10 journals.

**Table 1:** Journals with the most published papers.

Rank	Journal	Papers, n
1	World Neurosurgery	1096
2	Zhonghua Yi Xue Za Zhi	561
3	PLOS One	481
4	Molecular Medicine Reports	478
5	Oncotarget	474
6	Journal of Clinical Neuroscience	444
7	Oncology Letters	409
8	Chinese Medical Journal (English)	382
9	Journal of Neuro-Oncology	329
10	Clinical Neurology and Neurosurgery	327

### Institutions of Origin

Of scientific research institutions affiliated with the first author, 25.35% (4049/15,972) were from the top 10 institutions: Beijing Tiantan Hospital was the most prolific institution, with 985 papers (Table 2).

**Table 2:** Institutions with the most published papers.

Rank	Institution	Papers, n
1	Beijing Tiantan Hospital, Capital Medical University	985
2	Huashan Hospital, Fudan University	598
3	West China Hospital, Sichuan University	587
4	Jinling Hospital, Medical School of Nanjing University	388
5	Tangdu Hospital, Air Force Medical University	285
6	Peking Union Medical College Hospital	254
7	The General Hospital of Tianjin Medical University	248
8	Xijing Hospital, Fourth Military Medical University	240
9	Xiangya Hospital, Central South University	237
10	Changhai Hospital, Naval Medical University	227

### Co-word Analysis

A total of 24,469 keywords were extracted from the papers in the field of neurosurgery published by researchers in China from 1988 to 2018; 430 co-words had growth rates greater than 0 for 2015 to 2016, 2016 to 2017, and 2017 to 2018 (Multimedia Appendix 1). This filtering strategy was based on 430 pairs of co-words that can provide insight into emerging research subspecialties of great potential that have been attracting increasing attention within years and which might become the focus of neurosurgical research in the future.

### Biclustering Analysis of 430 Pairs

**Figure 3:**
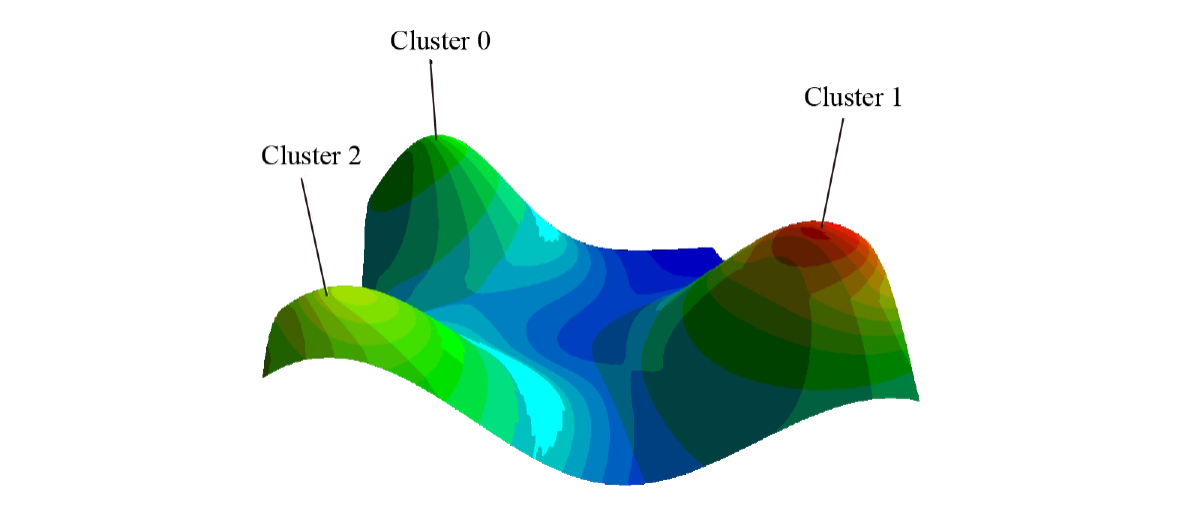
Mountain visualization of biclustering of the 430 pairs of co-words. Red represents a low standard deviation, and blue represents a high standard deviation among the co-words in the cluster.

**Figure 4:**
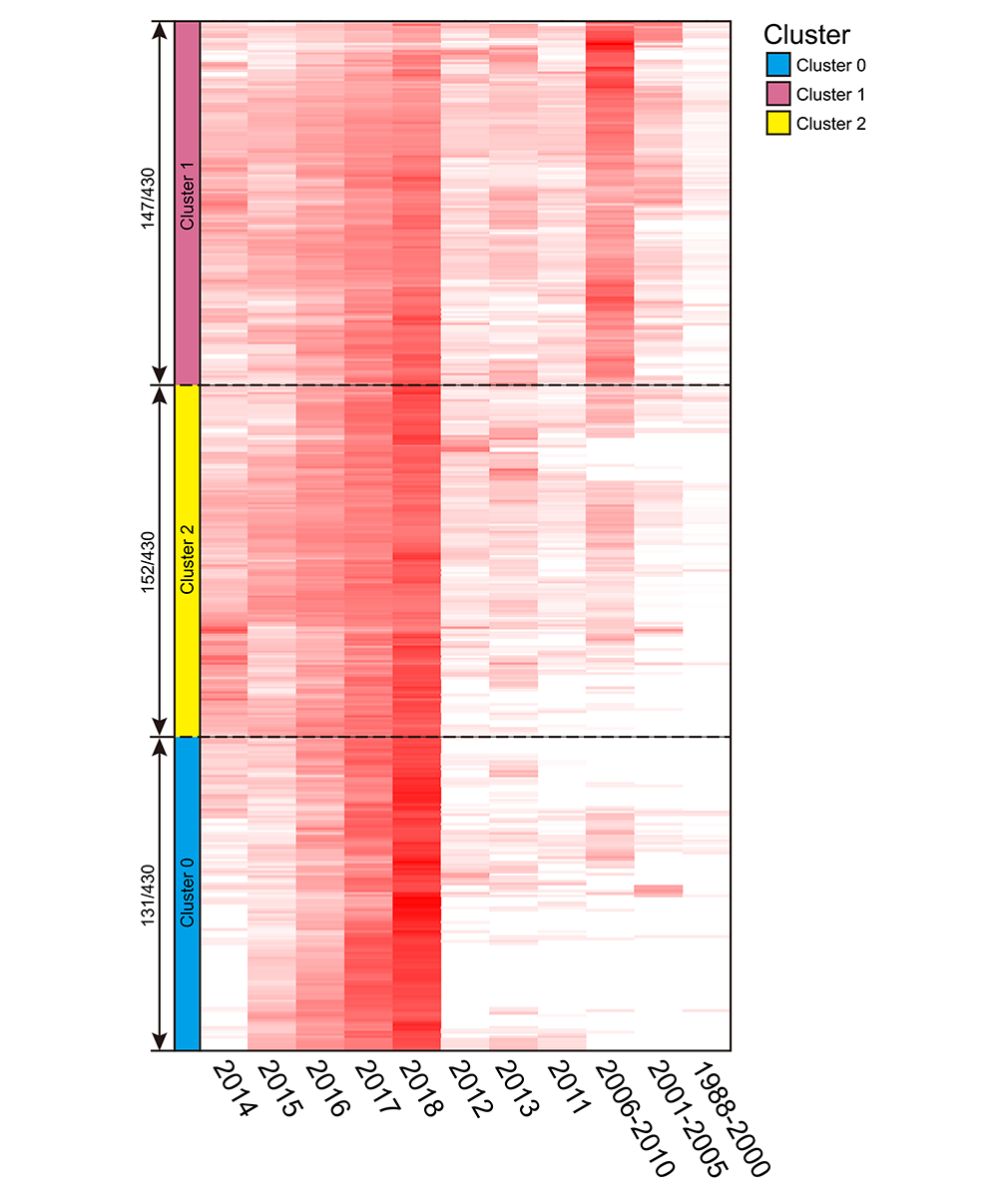
Heatmap of biclustering of the 430 pairs of co-words. Each row represents a pair of co-words, each column represents each stage of time, and the color of each box represents the frequency (the darker the red, the higher the frequency; white indicates that the frequency is close to zero [17]).

**Figure 5:**
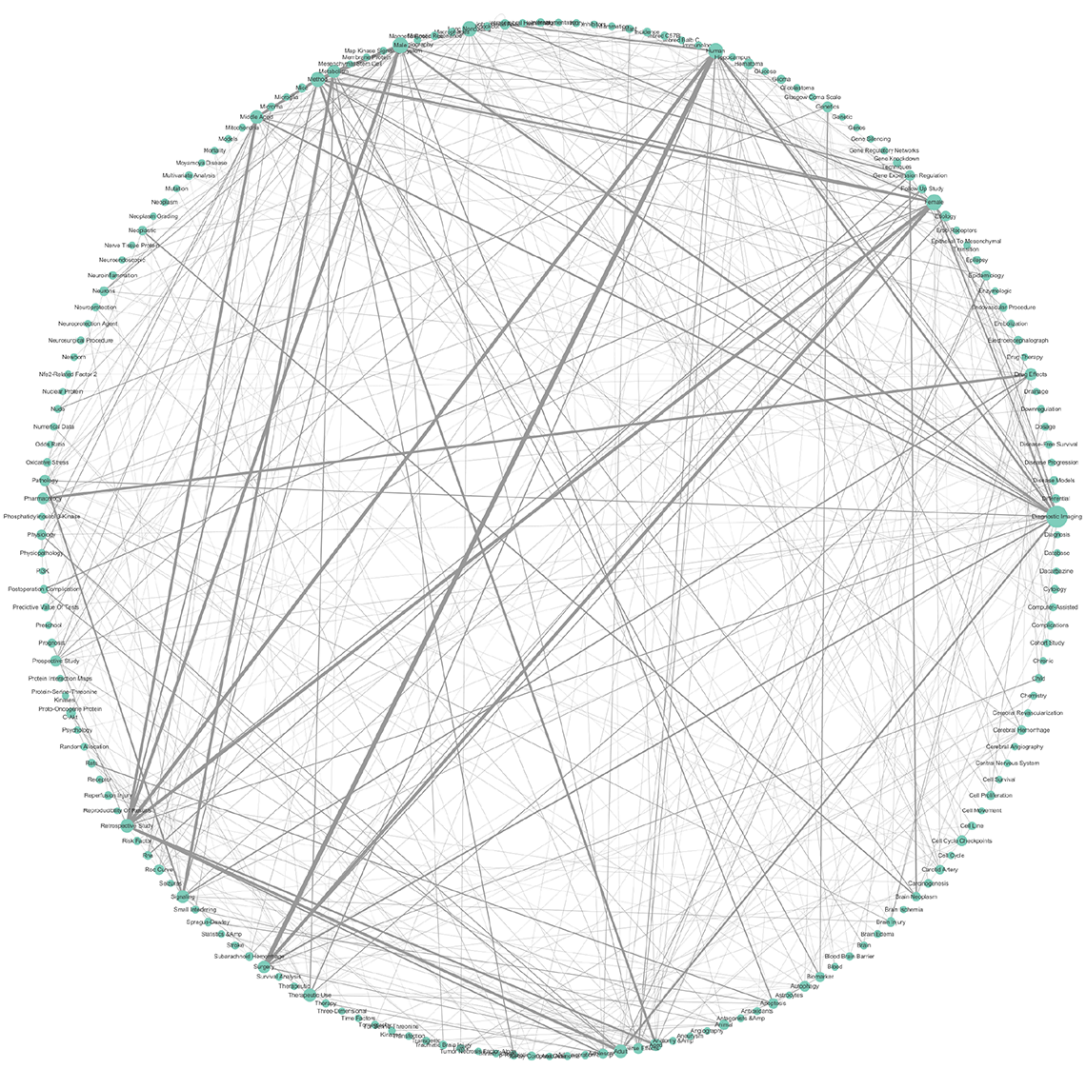
Relationships network among the 430 co-words. Each circle represents one keyword; the higher the frequency of coexistence, the larger the volume. Each line indicates a coexisting relationship between co-words (pairs of keywords); the higher the frequency of coexistence, the thicker the line.

The clustering results achieved after many attempts to obtain the best clustering strategy are presented in the form of a peak plot (Figure 3), with 3 clusters marked as peaks 0 to 2, and a heatmap (Figure 4), which is a schematic representation of high-dimensional data [15]. The height, volume, and color of each peak is proportional to the internal similarity within each cluster, the number of co-words in the cluster, and the standard deviation among the co-words in one cluster, respectively [16,17]. The independent distribution of peaks indicates that the clustering strategy has performed well [18]. Interrelationship networks between 430 pairs of co-words showed that *diagnostic image* coexisted the most with other keywords (Figure 5).

**Figure 6:**
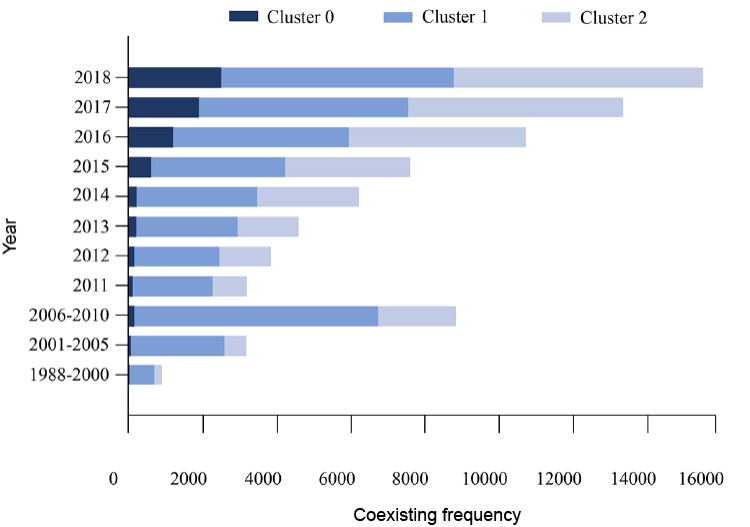
Temporal distribution of the co-word frequency for each cluster divided by stage.

The peak of Cluster 0 was the highest (internal similarity 0.945) has a large volume, which indicates that it contains a large number of co-words. The amount of papers containing the co-words in this cluster was low before 2015 (Figure 4 and Figure 6). Cluster 0 contained 131 co-words (total frequency: 7196/78,957, 9.11%). The most frequent co-words in Cluster 0 are (1) *diagnostic image* with *retrospective study* (428 papers), (2) *long noncoding* with *RNA* (208 papers), and (3) *long noncoding* with *human* (184 papers), respectively. These findings suggest that retrospective analyses of the imaging diagnosis of neurosurgical diseases and studies on human long noncoding RNA are leading areas in Cluster 0.

The peak of Cluster 1 was the lowest (internal similarity 0.916) and had the largest volume, with 152 co-words. Cluster 1 contained a large number of papers containing these co-words in this cluster published since 2001 (total frequency: 41331/78957, 52.35%). The most frequent co-words in Cluster 1 were (1) *surgery* with *human* (2357 papers), (2) *human* with *retrospective study* (1752 papers), and (3) *surgery* with *femal*e (1615 papers). These results suggest that literature on neurosurgical treatment, retrospective analysis, and surgical treatment of neurosurgical diseases of female patients occupy the main positions in Cluster 1.

The peak of Cluster 2 was medium height (internal similarity 0.931), indicating great similarities among co-words in this category, and of medium volume, with 147 co-words (total frequency: 30,430/78,957, 38.54%). The number of papers in this cluster has grown rapidly since 2014 and continues to increase rapidly. The top most frequent co-words in this cluster were (1) *metabolism* with *signaling* (1425 papers), (2) *drug effects* with *pharmacology* (1225 papers), and (3) *diagnostic imaging* with *male* (1093 papers). We concluded that, in Cluster 2, literature related to metabolic signaling pathways, pharmacology, and papers about neurosurgical imaging diagnosis of male patients were ranked in the leading position.

### Correlation Between the Number of Papers With Funding and Co-words

**Figure 7:**
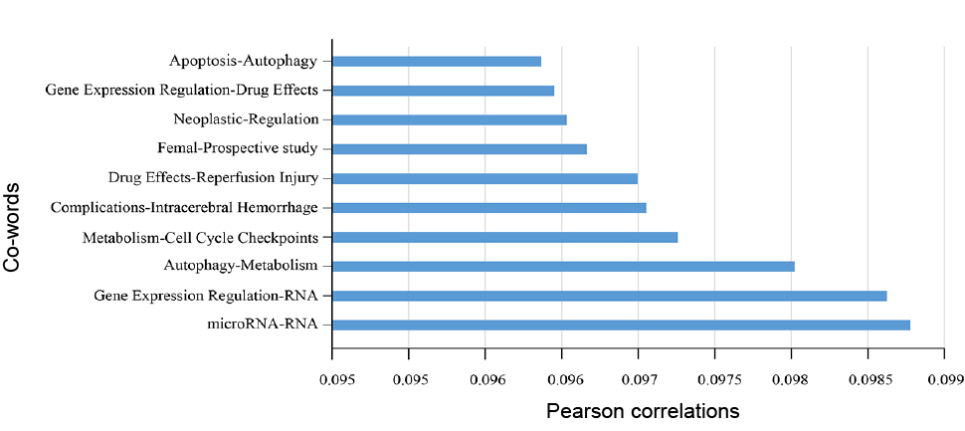
Correlation of the co-word frequency and number of papers with National Natural Science Foundation funds.

The distribution of National Natural Science Foundation of China funding for each topic of neurosurgical research represented by 430 co-words yearly (2011-2018) (Figure 7 and Multimedia Appendix 2). The topic *microRNA* was associated with the most papers funded by the National Natural Science Foundation of China (*r*=0.988), followed by *gene expression* with *RNA* (*r*=0.986) and *autophagy* with *metabolism* (*r*=0.980).

## Discussion

### Principal Findings

In this study, a bibliometric method, with keywords representing neurosurgery research papers from institutions in China published from 1988 to 2018 in the PubMed database, was used to analyze information on emerging topics. Quantitative analysis based on co-word and biclustering analyses of keywords in papers revealed development trends for each cluster of co-words and provides prospects for future research directions.

The bibliometric approach can help researchers address enormous amounts information when carrying out scientific investigations. The identification of potential research topics can promote academic advances in certain specialties and can help researchers grasp which emerging themes may become future directions of vital importance in certain disciplines [19].

In China, compared with the development of other fields, neurosurgery developed relatively late. Our study demonstrated a fluctuating and gradual increase in the amount of papers from China’s neurosurgical institutions over time. The amount of publications showed a noticeable increase in recent years, with an explosive increment in 2014 that accounted for 23% (5021/15,972) of the total number of papers published from 1988 to 2018. While the number of publications showed an overall upward trend, the period from 1988 to 2006 exhibited steady growth, and the period from 2007 to 2018 exhibited rapid growth; the number of published papers in the latter period was 19 times higher than that in the former. The amount of papers from journals in the SCI increased over time, accounting for 61.00% (9743/15,972) of the total number of papers, which indicates development of the research and the influence of the field of neurosurgery in China.

The keywords of papers are representative [20]. To a large extent, keywords can provide insight into the main research contents of the literature accurately and briefly [21]. The accurate extraction of publication keywords is a very important technology in the field of scientific literature searching and data mining. At present, when extracting keywords from literature and establishing a keyword database, the manual method often obtains the best results [22]. We standardized and filtered the extracted keywords of papers and established a keyword database for papers published by neurosurgical institutions in China from 1988 to 2018 based on PubMed data.

The biclustering approach, in contrast to traditional clustering approaches, can simultaneously cluster year of publication and co-words into matrix columns and rows, respectively. Cluster 0, which contains topics that began to gradually develop in recent years, contained the least co-words. This finding reveals that topics represented by co-words included in Cluster 0 as emerging themes may grow in importance in the future, given that they appeared with increasing frequency in recent years. Retrospective analyses of diagnostic imaging data can, not only assist in clinical decision making, but also, improve the accuracy of neurosurgical disease diagnosis and predict response to disease treatment [23,24]. Long noncoding RNA plays an important role in the development of tumors. With advances in sequencing technology, many new types of long noncoding RNA, which cannot be translated into proteins, have been discovered in the nucleus and cytoplasm of human tumor cells, usually ranging from 200 nucleotides to 100 kilobases [25,26]. Long noncoding RNA can carry out many functions, such as cell growth, development, senescence, and death, by regulating gene expression. At present, an increasing number of studies have shown some long noncoding RNA are closely correlated with the existence and development of tumors [27].

Co-words contained in Cluster 1 and Cluster 2 exhibited large cumulative frequency counts, representing topics that have been studied much in the past, and the amounts have shown growth in recent years. As evidenced by Cluster 1, surgery is still the first choice for neurosurgery-related diseases, such as gliomas. However, traditional craniotomy is dependent on operator experience and visual judgment, and its application is limited because of the inevitable damage to normal tissue and blood vessels [28]. With the help of diagnostic imaging to accurately resect lesions, microscopic resection, which can significantly improve clinical outcomes, has gradually become a main method of surgical treatment for human craniocerebral diseases [29,30]. As evidenced in Cluster 2, metabolic reprogramming of some craniocerebral tumors is closely related to signaling pathways, such as Wnt/β-catenin signaling pathways [31]. Metabolic changes in tumor cells are also intricately related to their characteristics, such as autonomous growth, antiapoptosis, unlimited proliferation, and angiogenesis [32].

Although the cumulative frequency counts of co-words in Cluster 0 were low, they increased from 2015 to 2018, which shows great potential for development; therefore, to a large extent, the topics in this cluster may become the future hotspots to which researchers should pay attention.

In general, funding facilitates scientific research. As the largest funding agency of natural science of China, the National Natural Science Foundation has made great efforts in encouraging the development of neurosurgical research over the past years. This study identified that the number of papers represented by the co-word pair *micro-RNA* and *RNA*, the co-word pair *gene expression regulation* and *RNA*, and the co-word pair *autophagy* and *metabolism* was proportional to the number of papers with National Natural Science Foundation in China funding, which may indicate that funding on these topics has accelerated research progress and literature output. Consistent co-word and biclustering analyses results suggest that research on the function of gene expression regulation of RNA, especially noncoding RNA such as micro-RNAs and long noncoding RNA, is of considerable importance and provide insight into future potential research directions.

Scientometric analysis can provide insight into the development of a research field, highlight research trends over time, and help identify valuable future directions. This study used bibliometric analysis to count and analyze the literature published by Chinese neurosurgical research institutions in the PubMed database from 1988 to 2018. Co-words were divided into 3 clusters that reveal current and future trends for neurosurgery research in China.

### Limitations

This study has some limitations. First, we focused only on papers that were indexed in PubMed (a freely available database). Although we believe that PubMed is a sufficiently large database for publications from neurosurgical institutions in China, other databases, such as Web of Science, should be used in future research to obtain more information such as number of citations. Second, the search string was relatively general and may not be able to identify all papers on neurosurgery in China. In addition, because the number of studies has increased over time, the numbers of papers published in recent years may have had an effect on our results to some extent. Future investigations should be performed to provide a more comprehensive evaluation of papers on neurosurgery in China.

### Conclusions

We applied bibliometric methods (co-word biclustering analysis and visual analysis) to examine research progression, emerging topics, and future potential research directions in the field of neurosurgery research in China. These findings can be used by neurosurgery researchers as a reference for choosing scientific research projects.

## Appendix

Multimedia Appendix 1 Temporal distribution of the 430 pairs of co-words.

Multimedia Appendix 2 Temporal distribution of funding from the National Natural Scientific Foundation.

## Abbreviations

SCI: Science Citation Index
